# Real-time fMRI neurofeedback compared to cognitive behavioral therapy in a pilot study for the treatment of mild and moderate depression

**DOI:** 10.1007/s00406-022-01462-0

**Published:** 2022-07-30

**Authors:** Mikhail Ye. Mel’nikov, Dmitriy D. Bezmaternykh, Andrey A. Savelov, Evgeniy D. Petrovskiy, Lyudmila I. Kozlova, Kira A. Natarova, Tatiana D. Larina, Tatiana M. Andamova, Mikhail Zvyagintsev, Mark B. Shtark, Klaus Mathiak

**Affiliations:** 1 Federal Research Center of Fundamental and Translational Medicine, Research Institute of Molecular Biology and Biophysics, Russian Federation, 2 Timakova str., Novosibirsk, Russia; 2 International Tomography Center, Russian Federation, 3a Institutskaya str., Novosibirsk, Russia; 3 International Institute of Psychology and Psychotherapy, Russian Federation, 2 Serebrennikovskaya str., Novosibirsk, Russia; 4 State Novosibirsk Clinical Psychiatry Hospital №3, Russian Federation, 2 Vladimirovskaya str., Novosibirsk, Russia; 5 RWTH Aachen University, Universitätsklinikum Aachen, 30 Pauwelsstrasse, Aachen, Germany

**Keywords:** Real-time functional magnetic resonance imaging neurofeedback (rt-fMRI NFB), Cognitive behavioral therapy, Depression, Medial prefrontal cortex, Learning curves

## Abstract

Real-time functional magnetic resonance imaging (rt-fMRI) neurofeedback was found to reduce depressive symptoms. However, no direct comparison of drug-free patients with an active psychotherapy control group is available. The present study compared rt-fMRI neurofeedback with cognitive behavioral therapy, as the standard treatment in patients declining anti-depressants. Twenty adult, drug-free patients with mild or moderate depression were non-randomly assigned either to a course of eight half-hour sessions of neurofeedback targeting the left medial prefrontal cortex (*N* = 12) or to a 16-session course of cognitive behavioral therapy (*N* = 8). Montgomery–Asberg Depression Rating Scale was introduced at baseline, mid-treatment, and end-treatment points. In each group, 8 patients each remained in the study to a mid-treatment evaluation and 6 patients each to the study end-point. ANOVA revealed a depression reduction with a significant effect of Time (F(3,6) = 19.0, *p* < 0.001, η^2^ = 0.76). A trend to greater improvement in the cognitive behavioral therapy group compared to neurofeedback emerged (Group × Time; *p* = 0.078). Percent signal change in the region of interest between up- and down-regulation conditions was significantly correlated with session number (Pearson’s *r* = 0.85, *p* < 0.001) indicating a learning effect. As limitations, small sample size could lead to insufficient power and non-random allocation to selection bias. Both neurofeedback and cognitive behavioral therapy improved mild and moderate depression. Neurofeedback was not superior to cognitive behavioral therapy. Noteworthy, the neurofeedback training course was associated with continuous improvement in the self-regulation skill, without plateau. This study delivers data to plan clinical trials comparing neurofeedback with cognitive behavioral interventions.

## Introduction

Depression is associated with alterations of function in various brain regions [8, 18, 36]. Anti-depressant medication and psychotherapy are the two major treatment strategies with comparable effectiveness [25]. However, attitudes of patients may favor treatment choices like bio- and neurofeedback [10, 27], approaches considered self-help and training may improve the feeling of self-efficacy [19, 34, 43], lower stigmatization [44], and exhibit only few (if any) adverse effects [32, 39]. In the field of the depression treatment, feedback methods training the modulation of heart rate variability [23, 24] or EEG frontal alpha asymmetry [3, 35, 38, 45] were followed by the current real-time fMRI neurofeedback (rt-fMRI NFB) targeting circumscribed brain structures [41].

Different protocols for rt-fMRI NFB were implemented in depression, e.g., upregulation of frontal activity related to processing positive emotions [12, 13]. Some reductions of depression symptoms after the protocol implementation were demonstrated [28, 34], though the more recent of these studies failed to demonstrate the specificity of this effect [34]. A protocol by Zotev et al. [52] is based on the left amygdala signal upregulation. In major depression, neural changes within the emotional network emerged [48–51] and depression reduction [49]. Recent proof-of-concept validated the hybrid fMRI-EEG neurofeedback [53]. Another study probed the salience network regulation in depressed patients [9]. Last, a pilot trial showed depression improvement after the training targeting the left dorsolateral prefrontal cortex [42].

One important feature of research in the field is a small number of sessions [6, 9, 28, 34, 42, 49, 50, 53]. This is related to the ability of rt-fMRI NFB to produce rapid effects compared to EEG neurofeedback [41] and partly to financial reasons. Thus, no data exist on the utility of courses longer than 5 sessions in depression.

Another noteworthy limitation on clinical evidence is that the controlled studies [9, 28, 34, 49, 50, 53] lack an active control condition necessary to understand the clinical utility of the method. In a recent publication [34], effect sizes of rt-fMRI NFB were matched to ones of other treatments indicating non-inferiority of rt-fMRI NFB. However, no direct comparison of the efficacy of rt-fMRI NFB to an active control condition is documented so far.

The aim of our study was to compare the effects of a course of rt-fMRI neurofeedback training encompassing 8 sessions with cognitive behavioral therapy in outpatients suffering from depression. The left medial prefrontal cortex (lMPC) served as target region since in this region chronic stress reduced dendritic density and in depressed subjects gray matter and dendritic spines were reduced, see [1]. Further, pilot data from our group found a robust involvement of the lMPC during rt-fMRI with positive vs neutral images from the EMOMadrid database [2] and faster learning of left as compared to right prefrontal neurofeedback in depression [17] corroborating a corpus of previous studies of left-lateralization of positive emotions (see review in [30]). As a general aim, we wanted to explore the time course of successful regulation and the involved neural structures.

## Methods

### Participants

Sixty-four participants (50 female) aged 18–55 (31.4 ± 9.0 yrs) were recruited via newspaper advertisement and psychotherapeutic office. Participants first underwent a psychiatric examination by trained psychotherapist (Kira A. Natarova) to establish the principle diagnosis of unipolar non-seasonal depressive disorder without psychotic features in accordance to ICD-10 criteria. Patients with other major psychiatric or neurological conditions or high risk of suicide were excluded. All patients reported no current psychotropic medication and were unwilling to receive anti-depressants.

The experiment was conducted in accordance with the Declaration of Helsinki. The study protocol was approved by the ethical commission of the Research Institute of Molecular Biology and Biophysics, which is currently a division of Federal Research Center of Fundamental and Translational Medicine. The approval carries the reference №1 from June 8, 2016. Prior written informed consent was obtained from all participants.

### Study overview

**Fig. 1:**
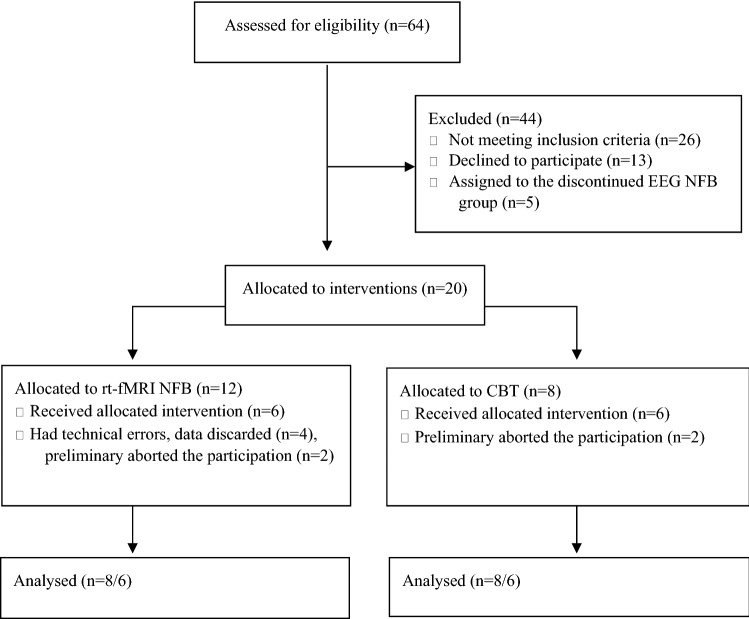
CONSORT flow diagram

The study was conducted from June 2017 until April 2019. A total of 64 candidates presented for the screening interview (see Fig. 1). They provided demographic data and were screened by a psychiatrist. For the reason that 25–50% of participants are known to fail sufficient self-regulation [6], we initially performed three sessions of EEG frontal alpha asymmetry neurofeedback to test the self-regulation abilities of the candidates. Only those who had a positive slope of frontal alpha asymmetry in at least 2 out of the 3 sessions or succeed in the 3rd session only, were included in the experimental cohort.

Thirteen candidates refused to participate before the interventions and 26 more demonstrated insufficient EEG self-regulation. The remaining 25 patients were non-randomly allocated to one of three treatment groups: fMRI neurofeedback, cognitive behavioral therapy, and EEG neurofeedback. The EEG neurofeedback arm (*n* = 5) was prematurely stopped due to a lack of voluntarily participating candidates; thus, all data of this group were discarded. Data of 4 participants were excluded due to technical issues. Of the remaining 16 participants, 8 underwent neurofeedback and 8 entered cognitive behavioral therapy (CBT) and received at least half of the course; 6 participants from each group completed the entire course.

The participants of both groups were tested at the course start, middle point, and the end-point. The neurofeedback course consisted of 8 sessions, whereas the psychotherapy course of 8 individual and 8 group sessions.

### Neurofeedback group

#### Procedure

Neurofeedback was performed at the International Tomography Center at Novosibirsk using a 3 T Philips Ingenia MR scanner. Each session consisted of two parts (the first one 15 min and the second one 10 min). In each odd sessions, both parts were using the neurofeedback condition, while in each even sessions, the second part was a transfer session without feedback signal.

Both, the neurofeedback and the transfer sessions comprised alternating blocks of up- and down-regulation of the hemodynamic activity in the target area, with a duration of 40 and 20 s, respectively. Participants were not provided with predetermined strategies, yet they were told that optimal strategies for upregulation would be related to positive emotions. In order to downregulate the target RoI emotionally, neutral strategies were recommended.

#### fMRI acquisition parameters

For structural reference, a T1-weighted isotropic 3d image was acquired using a turbo-field echo MR pulse sequence, (TR/TE = 7.5/3.7 ms, TI = 750 ms, voxel size 1 × 1 × 1 mm^3^). The functional T2*-sensitive images were recorded using echo-planar imaging (EPI; repetition time TR = 1,000 ms, matrix size 96 × 96, 16 sagittal slices, voxel size 2.3 × 2.3 × 6 mm^3^, flip angle FA = 70°, half scan (half-Fourier) option with the factor 0.7). Number of repetitions was 900 in the respective first or 600 in the second run per session. To account for T1 saturation, the first 5 volumes were discarded from analysis.

#### Online fMRI data analysis and experimental design

**Fig. 2:**
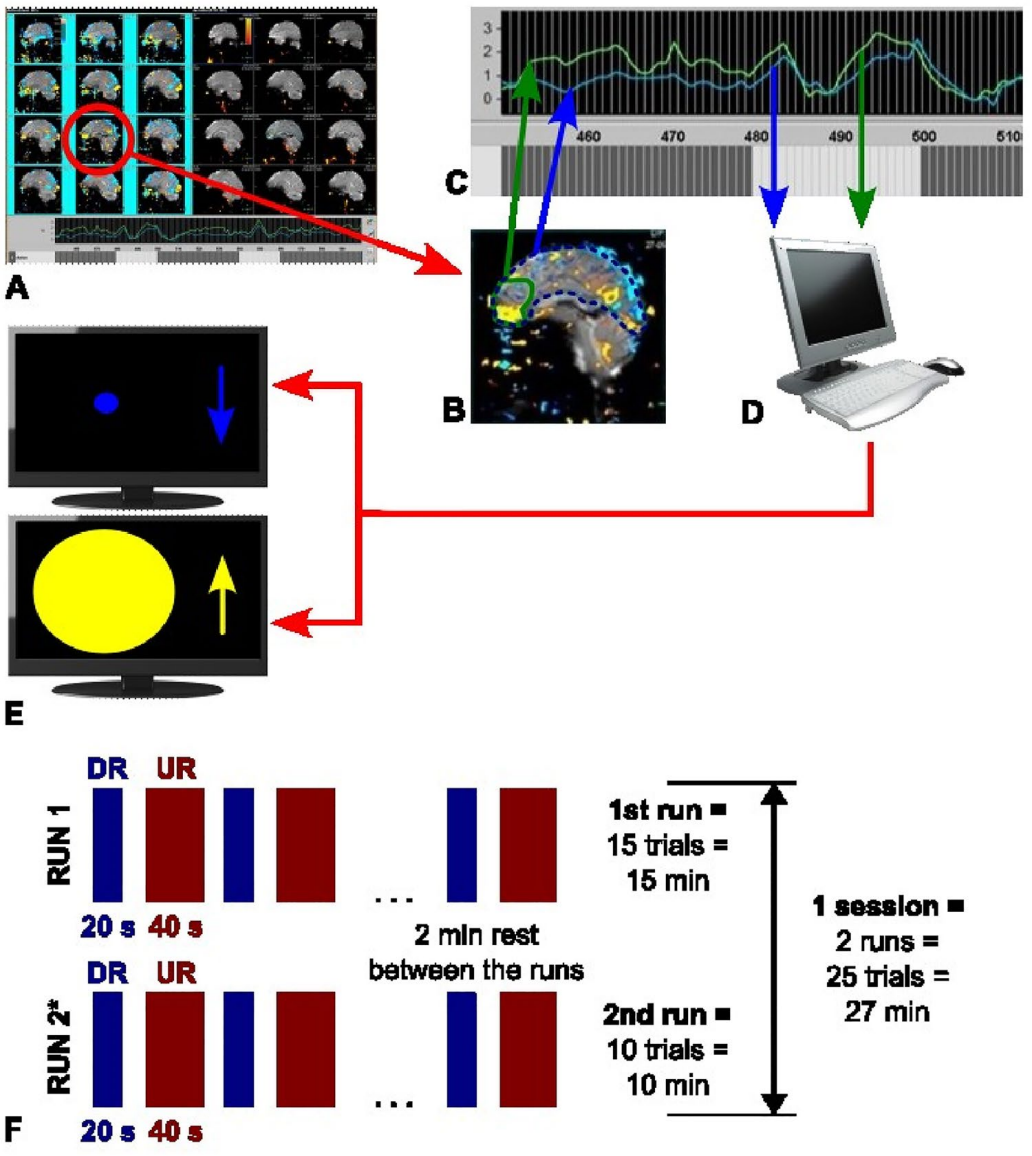
Schematic for dataflow and experimental design. **A** Screenshot from rt-fMRI software (Philips iViewBOLD). On the half-split screen, statistical maps for the contrast between up- and down-regulation are shown on the left and statistics on signal fluctuation for artifact control on the right. The time courses of percentage signal change is displayed on the bottom for both RoIs. **B** Enlargements of the selected RoIs in one slice (green solid line for RoI; blue dashed line for control RoI). **C** Enlargement of the signal time courses over the volume numbers (green: target RoI; blue control RoI). Below, the blocks mark the paradigm (dark: upregulation; light: down-regulation). **D** RoI data are extracted and exported to an external computer where signal changes in the target area over the control area are calculated. **E** This score is fed back to the participants as size of a disk. Upward arrow and yellow color indicated up-regulation (40 s each); downward arrow and blue color were shown during the down-regulation condition (20 s each). **F** Schematic of the experimental paradigm per each of the 8 neurofeedback sessions. The first run comprised 15 trials (20-s down-regulation and 40-s up-regulation each), the second run 10 trials. *Alternating, the second run was a transfer run with no feedback

Philips iViewBOLD software performed motion correction for inter-volume displacements, modeled the signal using a stair-step approximation of a standard hemodynamic response function, and calculated the BOLD signal from a 2D area on the 7th or 8th sagittal slice representing the left medial prefrontal cortex (lMPC) and from the full brain hemisphere volume within the same slice in percentage signal change. The regions of interest (RoIs) were marked manually by a trained operator (see Fig. 2A,B).

The activity curves were displayed as percentage signal change to the investigators on a iViewBOLD panel (Fig. 2C) and then captured by custom software and transferred to a separate PC (Fig. 2D). For baseline correction and to account for any global artifacts, the signal from the control area was subtracted from the one from the target area. This value was normalized for local minimum and maximum within the respective previous minute. The resulting score was fed back to the patients as a disk with smoothly changing size shown on an MR-compatible monitor (Fig. 2E). The delay from volume reconstruction to the visual feedback was below one TR (< 1 s). The screen also displayed a prompt indicating the block design of down- (20 s) versus the up-regulation condition (40 s) to the participants; run 1 comprised 15 and run 2 comprised 10 of these trials. In even sessions, no disk was shown during the second run providing a transfer condition without NFB (see Fig. 2F for the experimental design).

In parallel, MR data were stored on the scanner’s data base for subsequent offline processing. The MRI dataset is available at https://openneuro.org/datasets/ds003770/versions/1.1.0.

#### Offline fMRI data analysis

The image pre-processing was performed using the SPM 12 software (Wellcome Department of Imaging Neuroscience, London, UK). The first three volumes were discarded. Then slice timing, motion correction, and normalization into the MNI space (resampled voxel 2 × 2 × 2 mm) were conducted. The following processing was carried out in two ways. At first for a whole volume analysis, the functional volumes were smoothed using a Gaussian kernel with full width at half-maximum of 8 mm. For each session, the conventional GLM-analysis was applied. The resulting statistical parametric maps were obtained using t tests and corrected for multiple comparisons using FWE-correction at *p* < 0.05.

Second for RoI analysis, the normalized functional volumes were de-trended using Data Processing Assistant for Resting-State fMRI (DPARSFA). The RoI dynamics were extracted by intensity averaging. This allowed calculating percent signal change for each block with up-regulation compared with the down-regulation. Further, for the RoI difference, this parameter was averaged across blocks. Thus, the individual and group dynamics of regulation throughout the treatment course were computed. For a quasi-whole brain analysis, fixed-effect GLM was applied to the data of 6 participants who completed the whole course. Individual models implemented t contrasts averaging all 12 half-sessions. These contrasts were then processed with one-sample t test with respect to random effect.

### Cognitive behavioral therapy group

A psychiatrist and a clinical psychologist (Tatiana D. Larina and Tatiana M. Andamova) led the cognitive behavioral treatment groups of up to five patients at a time (8 sessions). The additional 8 treatment sessions followed a manual implemented at the institution based on the ABC model. The 8 consecutive group sessions were scheduled as 1. introduction; 2. ABC model, irrational beliefs; 3. beliefs leading to emotional responses; 4. cognitive bias, functional analysis; 5. cognitive bias and distortions (cont.); 6. cognitive reappraisal, ABCDE model; 7. assertiveness training; and 8. termination. Each session comprised individual case reports and homework assignments.

### Clinical assessment

The primary outcome measure was the Montgomery–Asberg Depression Rating Scale (MADRS) optimized for tracking the depression severity changes. The secondary measures were self-assessment scales, namely Beck Depression Inventory (BDI) and Zung Self-Rated Depression Scale (ZSRDS). Intelligence was measured with the Raven Progressive Matrices test. Edinburgh Handedness Inventory was used to establish the handedness.

Statistical analyses were performed using the IBM SPSS 21.0 software. Baseline equivalence of the groups was examined with chi-square test or t test. A repeated-measures ANOVA was applied to the psychometric data to test the effect of Time and the Group × Time (2 × 3) interaction with a posteriori Tukey test. Pearson’s correlation analysis assessed whether the fMRI signal values from the region of interest changed over time.

## Results

### Primary and secondary measures — clinical and psychological results

The descriptive statistics on demographic and baseline clinical characteristics of the groups are summarized in Table 1.

**Table 1:** Demographic data of the participants at baseline

Group	Rt-fMRI NFB	CBT	*﻿P*
*N*	8 (6 completed, 2 drop-outs)	8 (6 completed, 2 drop-outs)	n.s
Gender	8 females	5 females, 3 males	0.055
Age (Mean ± SD)	30.8 ± 10.3	30 ± 8.5	n.s
Handedness	2 left-handed	1 left-handed	n.s
Condition (ICD-X)	F32.0 (mild) – 2 F32.1 (moderate) – 6	F32.0 (mild) – 2 F32.1 (moderate) – 6	n.s
IQ, Raven (Mean ± SD)	96 ± 10	110 ± 11	0.035
MADRS (Mean ± SD)	27.3 ± 3.3	28.4 ± 2.9	n.s
BDI (Mean ± SD)	27.4 ± 12.7	24.1 ± 8.9	n.s
ZSRDS (Mean ± SD)	54.3 ± 5.7	48.3 ± 4.6	0.041

*N* number of participants in a group, *SD* standard deviation, *ICD-X* international classification of diseases, 10th revision, *IQ* intelligence quotient, *MADRS* Montgomery–Asberg Depression Rating Scale, *BDI* Beck depression inventory, *ZSRDS* Zung self-rating depression scale, *rt-fMRI NFB* real-time functional magnetic resonance imaging neurofeedback, *CBT* cognitive behavioral therapy, *p* statistical significance level, *n.s.* non-significant

**Fig. 3:**
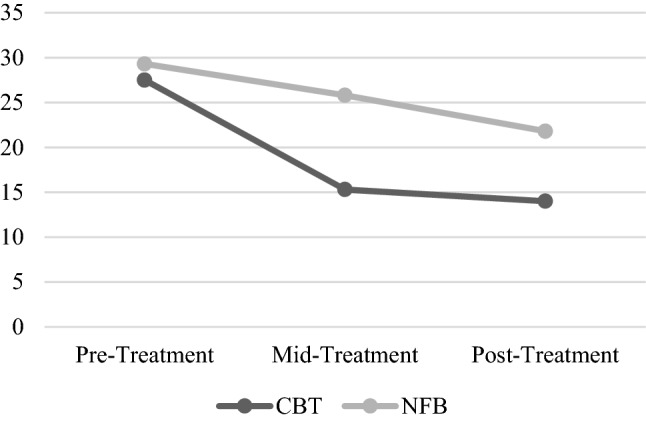
MADRS dynamics in the NFB and the CBT groups. The brighter line indicates the mean score of the NFB group, the darker line indicates the mean score of the CBT group

Repeated-measures ANOVA revealed a significant effect of Time and a trend for the Group × Time interaction on MADRS score (Table 2, Fig. 3). MADRS scores decreased from pre-treatment to middle-treatment and post-treatment points. The trend on Group × Time effect indicated a slightly more prominent MADRS decrease in CBT group (see Fig. 3). Hedge’s g’s for pre-to-post-treatment changes were g = –2.8 for the CBT group and g = –2.5 for the NFB group. Pre- to mid-treatment changes were g = –1.3 for the CBT group and g = –0.8 for the NFB group.

**Table 2:** ANOVA results for the depression estimates changes

	Time			Group x Time		
	F(3,6)	*p*	η^2^ (95% CI)	F	*p*	η^2^ (95% CI)
MADRS	19.0	0.0002	0.76 (0.34–0.85)	3.2	0.078	0.35 (0–0.58)
BDI	4.6	0.026	0.37 (0–0.58)	0.6	> 0.5	0.07 (0–0.29)
ZSRDS	1.3	> 0.3	0.16 (0–0.41)	0.3	> 0.5	0.05 (0–0.25)

*MADRS* Montgomery–Asberg Depression Rating Scale, *BDI* Beck depression inventory, *ZSRDS* Zung self-rating depression scale, *F* F-test value, *p* statistical significance level, *η*^2^ partial eta-squared, *CI* confidence interval

As concerns the secondary clinical outcomes, BDI score decreased significantly from pre-treatment to middle-treatment and post-treatment points (Table 2). No Time effect was revealed for ZSRDS. No Group x Time effect was evident for BDI or ZSRDS (see Table 2).

### Exploratory analysis — learning of self-regulation

**Fig. 4:**
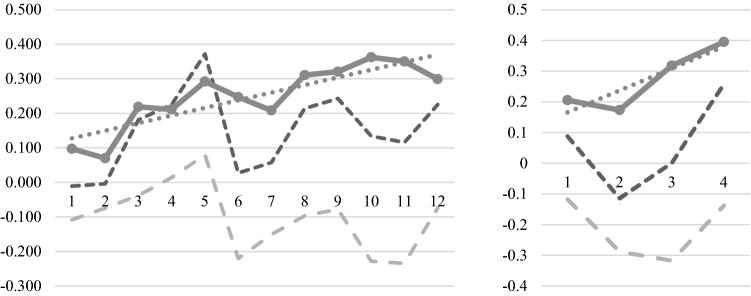
Average progress of the trainees in self-regulation learning. Left part illustrates half-sessions with feedback, right part depicts transfer half-session without a feedback. On the horizontal axis are half-session numbers and on the vertical axis are differences in fMRI signal between the up-regulation and down-regulation blocks, in percent. The solid gray line with markers illustrates feedback signal (difference between target and control RoIs). The thin dashed line is a linear trend for these dynamics. The dark dashed line is a target RoI, pale dashed line is a control RoI

The mean difference between the target and the control RoI in the six participants who finished the whole course correlated strongly with the half-session number (Pearson’s *r* = 0.85, *p* < 0.001; see Fig. 4). Separate analyses of the target and control RoI dynamics exhibited trends to the respective opposite directions (*r* = 0.33 and *r* = – 0.43, respectively). The curves were inter-correlated (*r* = 0.63) because the target RoI shared some non-specific activations (Fig. 4). The transfer sessions demonstrated a trend toward reinforced signal growth as well (Fig. 4), though the 4 time points were not enough for a meaningful correlation analysis.

### Exploratory analysis — FMRI BOLD activation map

The small sample size did not allow for thresholding at a significance level of *p* < 0.05 FWE-corrected. After pooling together the 12 half-sessions with feedback, the exploratory analysis at *p* < 0.001 with cluster size k > 10 revealed a small prefrontal activation cluster (peak t(5) = 26.8; peak MNI = [–2, 58, 26]; k = 11 voxels).

## Discussion

### General overview

Our study continues a line of research on the NFB for depression treatment by influencing the amygdala function either directly or via descending projections from the prefrontal cortex (see [22]) and specifically targeted downregulation of the amygdala by prefrontal cortex. We have chosen the left medial prefrontal cortex which is linked to emotional processing and exhibits a bias toward positive stimuli [5, 29].

Notably, we applied an active control condition, namely CBT to compare the clinical and psychological utility of these two approaches. CBT is a well-established and evidence-based treatment of depression [4, 26]. Succeeding a number of studies supported rt-fMRI superiority over the placebo training [9, 49, 50, 53], our study showing the relative place of the rt-fMRI in comparison with a ‘gold standard’ appeared timely. Given all rt-fMRI approaches lead to some modulation of the amygdala, our study provides important and open access pilot data for further controlled studies of the effects of different rt-fMRI protocols compared to those of the psychotherapeutic standards.

We applied a relatively long treatment course compared to the above mentioned trials. Thereby, we were able to assess a detailed learning curve. The continuously rising slope of the average percent of signal change from session to session supports the utility of long treatment courses. We suggest a combination with a longer follow-up assessment of the clinical catamnesis.

### Clinical and psychological results

In our pilot study, the MADRS score improved with time in both groups with rather large effect sizes and the effect size at the end-point was greater than at midpoint. However, the design of the study did not allow for direct testing the placebo reaction hypothesis. Nevertheless, Mehler et al. [34] suggested that such effect sizes are unlikely to be driven by a pure placebo effect. Indeed, treatment with sham whole-body hyperthermia led to moderate symptom improvements only [11]. Moreover, CBT has been consistently been shown to improve depression symptoms and it seems reasonable to assume a therapeutic effect in the CBT group [16]. Nonetheless, future study may consider including placebo and nocebo arms to differentiate spontaneous time and placebo effects on the time course of the depression symptoms in the target cohorts.

Given generally the good comparability of d and g effect size indices, the whole-course improvement on MADRS in our study was greater while the half-course improvement was lower than in the study by Young et al. [49]. Note that our study involved an 8-session course whereas Young’s comprised 2 sessions only. Similarly, when considering effect sizes of MADRS and HAM-D as similar, the interventions by Linden et al. [28] and Mehler et al. [34] led to a greater effect size than the half-course and to lower effect size at the end-point of our intervention. The percent of patients with relevant symptom reduction in our study was lower than in a previous study ([34]; 26% vs 42%). Remission rates in our study (1/8) were comparable with the one reported in ([28]; 2/8) but inferior to those in ([49], depending on criterion at 12/18 or 6/18). Thus, targeting the amygdala directly may be more effective than targeting the prefrontal cortex and, thus, could achieve stronger effects in the comparison with CBT than our protocol did. However, differences in recruiting approach, inclusion criteria, and allowance of concurrent medication also may account for the differences in outcomes. However, a recent study with a cross-over design for left or right lateral prefrontal cortex yielded in significant clinical improvements in 55% of the patients in a 4-week follow-up [17] which would be in line with the overarching importance of the temporal development for clinical improvements.

Large improvement on MADRS was accompanied by small (BDI) or no (ZSRDS) effect on the subjective measures. Such a dissociation may be related to the fact that MADRS is specially developed to trace changes in depression severity or to a possible mismatch between the clinician’s and patients’ own views on their symptoms. Noteworthy, the initial MADRS estimates were rather uniform which led to large effect sizes even with moderate improvements. Larger dispersion of the subjective measures led to the need for greater samples to notice moderate or small improvements.

### CBT vs rt-fMRI intervention

We found a trend to larger symptomatic improvement in the CBT group compared to the NFB group. Given the study was not adequately powered, this difference might become significant with a larger sample. Thus, CBT is a credible approach to a depression treatment [4, 26] and may in fact be more efficient than NFB. Therefore, a precise selection of patients must include personalized clinical profiles and patient preferences.

Another explanations for this unfavorable comparison can be a suboptimal NFB delivery in our study and inter-group differences in sex ratio, intelligence, and subjective depression in our study. Greater intelligence and lower subjective depression at baseline are probably related to receiving more benefits from the treatment [31] or the feedback modality should be improved to a more rewarding display (e.g., [33]).

In case CBT would be more effective than NFB, the usefulness of the latter as a means for depression treatment becomes questionable for general population. Indeed CBT shares some relevant features with NFB, e.g., the absence of the pharmacological side effects and the provision of skills and resources for self-help. CBT can be provided more readily and is less expensive than rt-fMRI neurofeedback. Therefore NFB applications may be limited to specific patient populations and research application, which should disentangle the underlying neural mechanisms [40].

### Benefits and the future role of rt-fMRI NFB

Possible benefits from using rt-fMRI NFB are shorter duration and the relative independence from a certain specialist and subjective therapeutic strategies. Likely, rt-fMRI NFB drives some positive long-term effects [37], yet their duration is still unknown. Further, fMRI neurofeedback may be an additional intervention to augment biological therapies or to enhance specific psychotherapeutic techniques, e.g., for cognitive reappraisal [54], and is an important addition to psychotherapy research methods.

Noteworthy, the rt-fMRI neurofeedback is a relatively young intervention is likely to evolve in future. Subsequent research may lead to a conclusive evidence on the most efficient neurofeedback targets (not limited to certain RoIs, but including functional connectivity, effective connectivity, network-related, and machine learning derived biomarkers). Further research may shed the light on the relative effectiveness of the neurofeedback performed during the resting state, symptoms provocation, including subconscious, and practicing normal cognitive operations disrupted in depression. The optimal feedback presentation method, strategy-related instruction, sessions number and duration, inter-session break duration, and home assignment issues are also subjects for ongoing and future studies. Altogether, these expected new data will improve the rt-fMRI neurofeedback effectiveness in depression. EFP-related algorithms reduce the neurofeedback expenses while probably preserving some benefits of the genuine fMRI-based training. Furthermore, with further technical progress, MR machines may become more readily available.

### FMRI BOLD data

In the mapping comparison for the regulation conditions, only in the prefrontal cortex a cluster emerged which was in line with the target region. Likely due to the small sample size, the underpowered design did not add sufficient information with respect to the neural mechanisms of neurofeedback learning or symptomatic improvements of depression treatments.

### Learning curves

The correlational analysis revealed self-regulation skill growth from session to session. The separate curves for target and control RoIs (yet failing significance) suggested that this skill included both increasing the prefrontal activity and decreasing the whole-brain activity in the up-regulation blocks compared to down-regulation blocks. The slope for the transfer sessions, in general, demonstrated the same tendency. Thus, the results of our study supported the improvement of the target RoI regulation from session to session, their specificity in terms of condition (up- and down-regulation), and brain area. Moreover, the data of transfer sessions suggest the generalizability of this skill to situations without feedback. As an alternative explanation of these data, the self-regulation improvement may have been a side effect of a partial spontaneous improvement frequent in mild and moderate depression [47].

### Limitations

We aimed to recruit patients with the first depressive episode unwilling to take medications and having the potential to rapidly master self-regulation via neurofeedback. These criteria reduced the number of available patients and might decrease the generalizability. The investigated samples are relatively small, yet still hold with previous patient studies using the demanding rt-fMRI NFB technique except a few more recent ones. Further limitations are that the group allocation was not randomized and study registration was performed after the finalization of the study due to its explanatory character. Since no placebo group was involved, non-specific biases may incur from placebo, nocebo, and Hawthorne effects as well as observation biases from spontaneous remissions and a regression to the mean. Future studies should rely on randomized controlled designs with predefined primary outcome measures to reduce the risk of bias (e.g., [7, 14, 17, 20, 46]. Further, active and inactive control conditions should be considered for a transparent description of therapeutic efficacy [15].

### Conclusion

We investigated the direct comparison of rt-fMRI neurofeedback to treat depressive psychopathology with the non-pharmacological gold-standard treatment, i.e., cognitive behavioral therapy. The depression symptoms improved in the neurofeedback and the psychotherapy group with large effect sizes in objective measures and a slight improvement in depression perception. A trend emerged for greater symptoms relief in the psychotherapy group. However, this study cannot provide conclusive evidence on this comparison. Thus, further research is warranted to the effectiveness of the rt-fMRI neurofeedback with other non-pharmacological interventions. Nevertheless, we could establish estimates for expected effects sizes and an ongoing training effect suggesting prolonged localized neurofeedback may achieve large effects in patients with light to moderate depression.

## Data Availability

The fMRI dataset generated during the current study along with demographical, clinical, and psychological measures is available in the openneuro.org repository, https://openneuro.org/datasets/ds003770/versions/1.1.0, while demographical, clinical, and psychological measures of both NFB and CBT patients are freely available in the osf.io repository, https://osf.io/qszd9/. The in-lab made software code is available from the corresponding author upon reasonable request.
